# Differences in wage rates for males and females in the health sector: a consideration of unpaid overtime to decompose the gender wage gap

**DOI:** 10.1186/1478-4491-11-9

**Published:** 2013-02-25

**Authors:** Nerina Vecchio, Paul A Scuffham, Michael F Hilton, Harvey A Whiteford

**Affiliations:** 1 Griffith Business School, Griffith Health Institute, Griffith University, 9726, Gold Coast, Queensland, Australia; 2 Centre for Applied Health Economics, School of Medicine, Griffith Health Institute, Griffith University, 4131, Logan, Queensland, Australia; 3 School of Population Health, The University of Queensland, Queensland Centre for Mental Health Research, 4076, Wacol, Queensland, Australia

## Abstract

**Background:**

In Australia a persistent and sizable gender wage gap exists. In recent years this gap has been steadily widening. The negative impact of gender wage differentials is the disincentive to work more hours. This implies a substantial cost on the Australian health sector. This study aimed to identify the magnitude of gender wage differentials within the health sector. The investigation accounts for unpaid overtime. Given the limited availability of information, little empirical evidence exists that accounts for unpaid overtime.

**Methods:**

Information was collected from a sample of 10,066 Australian full-time employees within the health sector. Initially, ordinary least-squares regression was used to identify the gender wage gap when unpaid overtime was included and then excluded from the model. The sample was also stratified by gender and then by occupation to allow for comparisons. Later the Blinder–Oaxaca decomposition method was employed to identify and quantify the contribution of individual endowments to wage differentials between males and females.

**Results:**

The analyses of data revealed a gender wage gap that varied across occupations. The inclusion of unpaid overtime in the analysis led to a slight reduction in the wage differential. The results showed an adjusted wage gap of 16.7%.

**Conclusions:**

Unpaid overtime made a significant but small contribution to wage differentials. Being female remained the major contributing factor to the wage gap. Given that wage differentials provide a disincentive to work more hours, serious attempts to deal with the skilled labour shortage in the health sector need to address the gender wage gap.

## Background

Although the difference between male and female hourly earnings has narrowed since the 1970s [1], studies on wages continue to report a persistent and sizable wage gap. Indeed, since 2005 evidence has emerged for a steady widening in gender wage differentials [2,3]. Several factors have been offered as explanations for the gap. Some factors relate to individual characteristics and work choices, others to institutional factors. Wages have been found to vary with the gender composition within occupations [4,5]. Rewards for human capital endowments, rather than differences in these endowments, offer another explanation for these gaps [6-8]. Other key determinants include industrial segregation, labour force history, under-representation of females with vocational qualifications and under-representation of females in large firms [3].

Compared with other OECD countries, Australian females possess a relatively small earnings disadvantage. This is mainly attributed to the comparatively high degree of labour market regulation [9]. Investigations reveal that, within Australia, gender wage differentials tend to be entrenched in the system of pay determination [10-13]. Sectors and occupations dominated by individual pay setting arrangements show a wider gender wage gap than those under a collective agreement. A larger gender wage gap exists among high-paid workers than low-paid workers [11], among the self-employed than wage/salary earners [9], and among the private sector than public-sector employees [10].

Typically, individuals achieve higher incomes by working more hours than their counterparts [14]. Advancement and promotion in the workplace is partly based on market signals such as positive unpaid overtime. Based on investigations of wage inequality between the United States and Germany, Bell and Freeman argue that labour supply decisions are forward looking and incentive driven [15]. Longer hours worked in one period improve the probability of promotion and improve the wage of workers in the future. Working longer hours is the most significant way to signal that an individual is a good (that is, quality) worker [15,16]. Higher-quality workers tend to be paid higher wages [17]. Various economic models offer explanations for wage differentials based on the quality of the worker [17]: firms may pay high wages to attract high-skill workers [18]; firms offer efficiency wages as an incentive for effort [19]; or workers bargain for a share of the gains from investments in their firm-specific skills [20].

Recognizing that wage differentials are partly the result of different hours of work, studies relating to wage gaps typically incorporate hourly wage into the analysis (for example, [7,10,14]). However, given the limited availability of information, little empirical evidence exists that accounts for unpaid overtime. Unpaid overtime is defined as any work undertaken in addition to ordinary or standard working hours in which an individual receives no compensation; for example, a health professional working 45 hours in a week but is paid for only 40 hours.

The Blinder–Oaxaca decomposition technique [21,22] addresses the ways in which different factors contribute to gender wage differentials. Previous studies that have examined the gender wage gap using the Blinder–Oaxaca decomposition method have reported a proportion of the gap explained by gender differences in human capital characteristics, job characteristics and family responsibilities. Yet an unexplained gap remains [9,23,24].

The aim of this study is to identify the magnitude of gender wage differentials among Australian employees within the health sector. This study takes advantage of new data that account for unpaid hours of market labour in the analysis of wage differentials. The Blinder–Oaxaca decomposition method is used to identify and quantify the contribution of individual endowments, and in particular unpaid overtime, to wage differentials. The hypothesis is that the sexual division of labour in the household [25] has led to females performing less unpaid overtime than males within the formal labour market. This hypothesis may partly explain the higher incomes of males relative to their female counterparts. Watson’s finding of a difference in the number of hours worked between male and female managers working full-time reinforces the argument that the domestic division of labour contributes to wage differentials [24].

In addition to fairness and equity there are strong economic imperatives for addressing the gender wage gap [3]. Using rigorous macroeconomic modelling techniques, researchers in Australia at the National Centre for Social and Economic Modelling concluded that the negative impact of the gender wage gap on performance stemmed primarily from the disincentives to work more hours [3]. Other researchers have found that as the gender wage gap decreases, females work more due to the added wage incentive [26]. This implies that cost of the gender wage gap on the health sector may be substantial. The demand for health services, partly driven by an ageing population, is expected to escalate. Workforce ageing and the greater feminization of some health professions have led to reductions in average hours worked, contributing to persistent labour shortages within the Australian health sector [27]. An understanding of the factors that contribute to wage differentials will assist agencies in implementing incentives to improve labour-force participation rates and increase hours worked.

## Methods

An econometric model for hourly wage is developed, and explanatory variables customary to studies of this nature are included in the log-lin model.

Hourly wage is determined in Equation (1) as:

(1) lnWi=β0+β1Genderi+β2UOi+βijXij+εi

where ln*Wi* is the log of hourly wage, *Gender* is a dummy variable with the value 1 for male, *UO*_*i*_ becomes unpaid overtime and *X*_*ij*_ becomes a vector of *j* variables known to impact on wage differentials that includes the level of education, occupation, experience, experience squared, marital status, number of children, number supervised, and private/public sector.

Initially, ordinary least-squares regression is used to identify the gender wage gap when unpaid overtime is included and then excluded from the model. As displayed in Equation (1), log of hourly wage is regressed against several variables characterizing individual and work characteristics. The gender dummy variable measures the gender differential. Later we stratify the sample by gender to allow a comparison between male and female incomes on the impact of unpaid overtime. Additional analysis estimates the hourly wage separately for each occupational group.

Lastly, the Blinder–Oaxaca decomposition [21,22] is employed to decompose the gap in outcomes between males and females. The gender pay gap in this log-lin method arises from two functions. The first is the actual difference in variables as shown by the difference in mean values between males and females – a part that is explained by group differences in productivity characteristics such as education, work experience and unpaid overtime. The second function is the discrimination function or unexplained residual – a part that cannot be accounted for by differences in characteristics [28]. This unexplained component is traditionally interpreted as a measure of discrimination. The expectation is that similar endowments should translate into similar wage levels among females and males. Any differences in male and female earnings from this function arise from the difference in male and female coefficients [29].

The difference in the gender wage gap (that is, Blinder–Oaxaca decomposition) arises from the following equation:

(2) $$\begin{array}{c} \ln\left(W^{M}\right)-\ln\left(W^{F}\right)=\left[\left(x_{\mathrm{ij}}{}^{M}-x_{\mathrm{ij}}{}^{F}\right)\;\beta_{\mathrm{ij}}{}^{M}\right]\\ \;+[\left(\beta_{\mathrm{ij}}{}^{M}-\beta_{\mathrm{ij}}{}^{F}\right)\;x_{\mathrm{ij}}{}^{F}\\ \;+\left(\beta_{0}{}^{M}-\beta_{0}{}^{F}\right)] \end{array}$$

where ln(*W*) is the log of hourly wages, *x*_*ij*_^*M*^ is the vector of means from the male equation, *x*_*ij*_^*F*^ is the vector of means for the female equation*, β*_*ij*_^*M*^ is the vector of coefficients from the male equation, and *β*_*ij*_^*F*^ is the vector of coefficients from the female equation.

A cross-sectional approach is used to investigate gender–wage differentials. Although cross-sectional analysis ignores the effects of institutional and technological change and changes in the labour market over time [30], it does permit the inclusion of certain variables, such as human capital, and allows an examination of the wage distribution across individuals [31]. Furthermore, the persistent nature of the gender wage gap shows that cross-sectional analysis is not as biased as it would be in other applications, since the wage gap shows little variation overtime [32].

### The sample

The data presented are subgroups of a larger study, the Work Outcomes Research Cost-benefit Project [33]. The information in this paper was collected from the Health and Performance at Work Questionnaire (HPQ) developed by the World Health Organization. Information about the HPQ can be accessedonline [34]. Employees over the age of 18 years were invited to respond to the HPQ. Participation in the survey was voluntary and confidential. The University of Queensland Human Research Ethics Committee approved the study protocol. The survey derived from the Australian workforce during 2005 and 2006 had a response rate of 25%.

The data used for the analysis were confined to Queensland employees working in the health sector, aged 25 to 64 years. In this paper the health sector includes those employed in the community sector who assist health professionals in the provision of patient care. This study follows the practice of most Australian studies of gender wage differentials in Australia by focusing on full-time workers [1,8-11,24]. Confining the analysis to workers in the health sector captured the award agreements of the state of Queensland and the industry. Isolating the sample to one industry in one Australian state also reduced the complexities associated with heterogeneous institutional factors and labour market forces experienced among various industries and Australian states.

Those aged 65 and over were excluded from the analysis because the minimum pension age for males is 65 years in Australia. Persons under 25 years old were also excluded because many had not yet completed their tertiary studies, which, by inclusion, would add greater uncertainty and heterogeneity to the sample. That is, no information was collected on the level and type of studies undertaken – if any – by this group.

After excluding those who did not fit this study’s criteria, 10,066 observations remained for analysis. A comparison of the Work Outcomes Research Cost-benefit dataset with the Australian Bureau of Statistics census data of 2005 [35] showed similar demographics for employees aged 25 to 64. Discrepancies in the percentage of full-time employees in the occupation categories between the two surveys are probably due to the slight differences in the categorization of workers.^a^

There are two potential sources of selection bias in the chosen sample. First, there is the decision to enter the paid labour market. Wages are only observed for people who are participating in the labour force and this might be a selective group. Second, there is the decision to work full-time or part-time conditional upon labour market entry. Several studies report that the correction for selection bias in the analysis of gender wage differentials, such as the Heckman procedure, has produced conflicting results and the application of this methodology may indeed introduce more bias (for a review of the literature, see [9]). Similar to Eastough and Miller [9], this study does not correct for selectivity bias that would require a more complex selection mechanism than the single selection mechanism.

### The data

The hourly wage rate was constructed by dividing the annual income by the hours employees were expected to work in a typical 7-day period, divided by 52 weeks. The variable was then converted to its logarithm. This produced a level of skewness and kurtosis within the acceptable range of a normal distribution.

The construction of the unpaid overtime variable involved several steps. The HPQ survey asked employees: ‘About how many hours altogether did you work in the past seven days?’. This information gave the actual hours worked over the week. Respondents were also asked: ’How many hours does your employer expect you to work in a typical seven-day week? (If it varies, estimate the average.)’. This information determined the expected hours worked.

Unpaid overtime was calculated by the actual minus expected hours worked per week.^b^ A positive (negative) sign indicated that the employee worked hours above (below) their employer’s expectation. If an employee was expected by their employer to perform a certain amount of overtime, then actual and expected hours would equal each other.

This indirect measure of unpaid overtime does have an advantage over directly asking employees to report the amount. Some employees may claim to work unpaid overtime even though their contracts do not specify the length of working hours. This is typical of managerial and professional occupations. In these cases it is more difficult to ascertain how and why it is believed that work has been undertaken for no pay [36]. The indirect estimate of unpaid overtime is useful when investigating a range of occupational groups. Hours worked beyond the expected level probably do not attract pay, even though there is no direct evidence of this. In contrast, supposing that an employee works ‘*x*’ hours of overtime and their employer expects them to work ‘*x*’ hours of overtime, then one would expect this to be reflected in the respondent’s annual income.

The data did not include information on actual labour market experience. In the absence of such information, the traditional approach is to use the Mincer proxy for potential labour market experience (that is, experience proxy, PE) calculated as age minus number of years of education minus 6.^c^ The derivation of this variable required a number of intermediate steps. To calculate the number of years of full-time equivalent education, it was assumed that each post-secondary qualification lasted a specific length of time [37]. Similar to other studies, PE and PE^2^ were also included in the model [9,32,37]. PE^2^ captured the effect of labour market experience on income. Following the usual practice (for example, see [9,32]), the analysis included the potential experience and added a children status variable to capture the effect of child-rearing on females’ labour force experience.^d^

## Results

### Descriptive statistics

Referring to Table 1, although both males and females are expected to work around 41 hours per week, the actual hours worked by males is greater (46 hours) than that worked by females (44 hours)*.* The hourly wage is A$28.70 for males and A$22.70 for females. This represents an unconditional hourly wage rate differential of 20.9%.

**Table 1:** Definition of variables

		**Pooled**	**Male**	**Female**
**Variable name**	**Definition of variable**	**%, mn (*n *= 10,066)**	**%, mn (*n *= 3,451)**	**%, mn (*n *= 6,615)**
Log of hourly wage	Continuous variable	3.1	3.3	3.1
Annual income	Continuous variable	51,615.6	60,590.4	46,933.6
Hourly wage	Continuous variable	24.8	28.7	22.7
Actual hours worked	Continuous variable	44.8	46.1	44.1
Expected hours worked	Continuous variable	40.6	40.9	40.5
Unpaid overtime^a^	Employee’s actual hours minus expected hours worked. Continuous variable	4.2	5.2	3.7
Gender (%)	1 male	34.3		
	0 female	65.7		
Marital status (%)	1 married/cohabitation – referent	71.1	79.6	66.7
	Never married	15.5	12.1	17.3
	Separated/divorced/widowed	13.4	8.3	16.0
Education (%)	Year 11 or under	17.1	16.5	17.4
	Year 12	7.5	6.8	7.9
	Tertiary education	23.4	23.9	23.1
	Degree graduate – referent	27.3	26.1	27.9
	Postgraduate	24.7	26.7	23.7
Occupation (%)	Manager – referent	11.2	13.0	10.3
	Professional/technical	61.1	58.9	62.2
	Clerical/service	23.1	16.1	26.7
	Trade/labour	4.6	12.1	0.7
Private/public sector (%)	Local – referent	16.5	30.0	9.4
	State	80.4	67.4	87.2
	Private	3.1	2.6	3.4
Supervision	Number of people personally supervise	6.0	6.6	5.6
Number of children	Continuous variable	0.5	0.7	0.4
PE	Labour market experience proxy. Continuous variable	22.7	23.3	22.3
PE^2^	Continuous variable	630.2	657.7	615.8

Full-time employees of the health sector. Mn represents mean values.; PE, experience proxy. ^a^Actual hours minus expected hours worked by employee.

Source: Work Outcomes Research Cost-benefit Survey 2005/06.

### Ordinary least-squares regression

The estimated coefficients and adjusted *R*^2^ statistics of the regression results for the pooled model and models stratified by gender and occupation are reported in Tables 2 and 3. The adjusted *R*^2^ statistics of the regression results for the pooled, male and female samples (see Table 3) are 0.428, 0.463, and 0.358, respectively.

**Table 2:** Regression results

	**Pooled (*n *=10,066)**				**Male (*n *= 3,451)**				**Female (*n *= 6,615)**			
	***B***	**SE**	**95% CI**		***B***	**SE**	**95% CI**		***B***	**SE**	**95% CI**	
(Constant)	3.043***	0.013	3.017	3.069	3.096***	0.025	3.044	3.148	3.082***	0.015	3.053	3.112
**Married:** Sepdivwid^a^	−0.021*	0.009	−0.039	−0.003	−0.055**	0.019	−0.094	−0.016	−0.007	0.010	−0.027	0.012
NvrMar^a^	0.005	0.009	−0.022	0.013	−0.037*	0.017	−0.073	−0.002	0.017	0.010	−0.002	0.037
**Education:**yr11/lower^b^	−0.288***	0.011	−0.311	−0.265	−0.304***	0.021	−0.347	−0.261	−0.275***	0.013	−0.302	−0.248
Yr 12^b^	−0.176***	0.013	−0.202	−0.150	−0.194***	0.024	−0.244	−0.144	−0.162***	0.015	−0.191	−0.132
Tery ed^b^	−0.166***	0.009	−0.184	−0.148	−0.173***	0.016	−0.205	−0.140	−0.158***	0.011	−0.179	−0.136
Post grad^b^	0.106***	0.008	0.090	0.123	0.168***	0.015	0.138	0.197	0.067***	0.010	0.048	0.087
**Occupation:** Manager^c^	0.077***	0.010	0.057	0.097	0.055**	0.017	0.021	0.089	0.088***	0.012	0.064	0.112
Clericalservice^c^	−0.166***	0.009	−0.183	−0.149	−0.203***	0.017	−0.238	−0.168	−0.163***	0.010	−0.183	−0.144
Trade/labour ^c^	−0.343***	0.016	−0.376	−0.310	−0.304***	0.021	−0.347	−0.261	−0.381***	0.042	−0.464	−0.297
**Number of children**	0.069***	0.003	0.063	0.075	0.070***	0.005	0.060	0.080	0.068***	0.004	0.061	0.076
**Govt:** local^d^	0.116***	0.009	0.098	0.134	0.065***	0.013	0.039	0.091	0.172***	0.012	0.147	0.196
Private^d^	−0.030	0.017	−0.064	0.003	0.028	0.033	−0.039	0.095	−0.060**	0.019	−0.098	−0.022
**Number supervised**	0.002***	0.000	0.001	0.002	0.002***	0.000	0.001	0.007	0.002***	0.000	0.001	0.002
**PE**	0.005***	0.001	0.002	0.007	0.014***	0.002	0.010	0.019	0.001	0.001	−0.002	0.004
**PE**^**2**^	0.000**	0.000	−0.000	−0.000	−0.000***	0.000	−0.000	−0.000	−0.000	0.000	−0.000	0.000
**Unpaid overtime**	0.007***	**0.000**	**0.007**	**0.008**	0.008***	**0.001**	**0.007**	**0.009**	0.007***	**0.000**	**0.006**	**0.008**
**Gender**	0.154***	**0.007**	**0.141**	**0.167**								
Adj *R*^2^	*0.428*				*0.463*				*0.358*			
**Gender**^**e**^	0.163***	**0.007**	**0.150**	**0.177**								
Adj *R*^2^	*0.407*											

Impact of unpaid overtime on income, pooled sample and stratified by gender. Dependent variable is log of hourly wage. CI, confidence interval; PE, experience proxy; SE, standard error. ^a^Referent is married. ^b^Referent is degree graduate. ^c^Referent is professional/technical. ^d^Referent is state. ^e^Model without unpaid overtime variable. ***P ≤0.001, **P ≤0.01, *P ≤0.05.

Source: Work Outcomes Research Cost-benefit Survey 2005/06.

**Table 3:** Regression model

	** Manager (*n *= 1,128)**			**Professional/technical (*n *= 6,151)**			** Trade/labour (*n *= 463)**			**Clerical/service (*n *= 2,324)**		
	*** B***	** 95% CI**		*** B***	** 95% CI**		*** B***	** 95% CI**		*** B***	** 95% CI**	
(Constant)	3.144***	3.044	3.243	3.046***	3.015	3.078	2.570***	2.345	2.795	2.897***	2.820	2.971
**Married:** Sepdivwid^a^	−0.038	−0.090	0.014	−0.031*	−0.056	−0.007	−0.017	−0.096	0.062	0.010	−0.021	0.041
NvrMar^a^	0.045	−0.014	0.105	−0.025*	−0.047	−0.003	−0.015	−0.091	0.061	0.047*	0.011	0.083
**Education:** yr 11/lower^b^	−0.389***	−0.455	−0.322	−0.334***	−0.370	−0.298	−0.153*	−0.299	−0.006	−0.190***	−0.239	-0.142
Yr 12^b^	−0.216***	−0.296	−0.135	−0.176***	−0.215	−0.136	−0.103	−0.262	0.057	−0.107***	−0.158	-0.057
Tery ed^b^	−0.199***	−0.250	−0.149	−0.167***	−0.190	−0.143	−0.107	−0.257	0.042	−0.104***	−0.150	-0.058
Post grad^b^	0.078**	0.032	0.125	0.106***	0.087	0.125	−0.183	−0.503	0.137	0.031	−0.065	0.126
**Number of children**	0.083***	0.068	0.098	0.065***	0.057	0.073	0.106***	0.065	0.147	0.066***	0.0568	0.077
**Govt:** local^c^	0.169***	0.118	0.220	0.078***	0.051	0.104	0.079**	0.024	0.134	0.164***	0.135	0.194
Private^c^	−0.134*	−0.238	−0.030	−0.036	−0.080	0.008	0.143	−0.146	0.433	0.023	−0.039	0.084
**Number supervised**	0.001*	0.000	0.002	0.002***	0.002	0.003	−0.002	−0.005	0.001	0.002**	0.001	0.003
**PE**	0.007	−0.002	0.015	0.003*	0.000	0.006	0.007	−0.006	0.020	−0.001	−0.006	0.005
**PE**^**2**^	−0.000	−0.000	0.000	−0.000	−0.000	0.000	−0.000	−0.000	0.000	−0.000	−0.000	0.000
**Unpaid overtime**	0.006***	**0.004**	**0.008**	0.007***	**0.007**	**0.008**	0.008***	**0.006**	**0.011**	0.008***	**0.006**	**0.010**
**Gender**	0.147***	**0.109**	**0.184**	0.177***	**0.160**	**0.195**	0.170***	**0.091**	**0.250**	0.089***	**0.062**	**0.116**
Adj *R*^2^	0.407			0.302			0.228			0.235		
**Gender**^d^	0.149***	**0.111**	**0.188**	0.190***	**0.172**	**0.207**	0.179***	**0.095**	**0.262**	0.094***	**0.067 0.122**	
Adj *R*^2^	*0.390*			*0.276*			*0.156*			*0.206*		

Impact of unpaid overtime on income, stratified by occupation. Dependent variable is log of hourly wage. CI, confidence interval; PE, experience proxy. ^a^Referent is married. ^b^Referent is degree graduate. ^c^Referent is state. ^d^Model without unpaid overtime variable. ***P ≤0.01, **P ≤0.05, *P ≤0.10.

Source: Work Outcomes Research Cost-benefit Survey 2005/06.

### Pooled sample

The results presented in Table 2 for the pooled sample show that income rises with each incremental increase in educational attainment. Managers followed by professional/technical occupations earn higher incomes than the remaining occupational categories. A greater number of children is associated with higher incomes. Public-sector employees are better remunerated than their private-sector counterparts.

Referring to the coefficient estimate for unpaid overtime, earnings tend to be higher for individuals working beyond the expected hours of work. (Note: if workers are paid for overtime, then this is not considered as working beyond the expectations of their employer). An increase of 1 hour in average unpaid overtime results in a 0.7% increase in the average hourly wage rate.

To obtain the relative change in mean income for gender, we take the antilog of the estimated dummy coefficient and subtract it from 1 [38]. The mean income of females becomes 16.6% lower than that of males. When unpaid overtime is excluded from the model, the mean income of females is 17.7% lower than that of males (antilog of 0.163).

### Ordinary least squares: sample stratified by gender

The rationale for the stratification by gender is that there are significant differences between males and females in the determinants of earnings that cannot be captured by a simple gender dichotomous variable. After controlling for other variables, the analysis shows that the impact of unpaid overtime on income is slightly more responsive in the male than the female sample (see Table 2). In the male sample, when average unpaid overtime increases by 1 hour, the hourly wage rate increases by 0.8% on average. For females, the associated increase is 0.7%. The remaining coefficient estimates are similar across the two samples and possess the expected signs. Males receive higher returns on schooling than females, and married males receive significantly higher wages than single and separated males.

### Ordinary least squares: sample stratified by occupation

Stratifying the sample by occupation indicates that the gender wage gap varies across occupations (Table 3). Holding other variables constant, the mean gender wage gap is: manager, 15.8%; professional/technical, 19.4%; trade/labour, 18.5%; and clerical/service, 9.3%. Across all occupations the gender wage gap increases slightly when unpaid overtime is excluded from the model (16.1%, 20.9%, 19.6% and 9.9%, respectively). The remaining coefficient estimates are similar across the four samples and possess the expected signs.

### Blinder–Oaxaca decomposition

The results presented in Tables 2 and 3 reveal that male earnings are greater than female earnings after adjusting for characteristics. The analysis from the Blinder–Oaxaca decomposition reveals the relative proportions of the gender wage gap that can be attributed to differences in characteristics and differences in the parameters. For ease of interpretation the Blinder–Oaxaca decomposition results are retransformed from the logarithmic scale (log of wages is the dependent variable) to the original scale of hourly wages.

The first three rows of Table 4 present the decomposition output of the mean predictions by groups and their difference. The mean hourly wage is A$25.95 for males and A$21.27 for females, yielding a raw gender wage gap differential of 22.0%. Adjusting female’s endowment levels to the levels of males would increase wages by 4.6%. A gap of 16.7% remains unexplained.

**Table 4:** **Blinder–Oaxaca decomposition of differences in hourly wage rates between males and females**^**a**^

**Overall**	**Coeff.**	**Robust SE**	**95% CI**		**Coeff.**	**Robust SE**	**95% CI**	
group_1 (males)	25.948	0.192	25.574	26.328				
group_2 (females)	21.274	0.093	21.092	21.456				
difference	1.220	0.010	1.199	1.240				
explained	1.046	0.006	1.034	1.058				
unexplained	1.166	0.008	1.150	1.183				
	** Explained**				** Unexplained**			
Separated/div/wid	1.001	0.001	1.000	1.003	0.995	0.002	0.991	1.000
Never married	1.000	0.000	0.999	1.001	0.992	0.003	0.987	0.998
Year 11/lower	1.002	0.002	0.998	1.007	0.995	0.004	0.987	1.003
Year 12	1.002	0.001	1.000	1.004	0.998	0.002	0.993	1.002
Tertiary	0.999	0.001	0.996	1.002	0.997	0.004	0.988	1.005
Postgraduate	1.003	0.001	1.001	1.005	1.026	0.005	1.016	1.036
Manager	1.002	0.001	1.001	1.003	0.996	0.003	0.991	1.001
Clerical/service	1.018	0.002	1.015	1.021	0.993	0.004	0.986	1.000
Trade/labour	0.962	0.002	0.957	0.967	1.005	0.002	1.002	1.008
Number of children	1.018	0.002	1.014	1.021	1.001	0.004	0.994	1.008
Local	1.024	0.002	1.020	1.028	0.980	0.003	0.973	0.986
Private	1.000	0.000	1.000	1.001	1.003	0.001	1.000	1.005
Number supervised	1.002	0.001	1.001	1.003	1.001	0.003	0.995	1.007
PE	1.005	0.001	1.002	1.008	1.355	0.079	1.208	1.520
PE^2^	0.997	0.001	0.994	1.000	0.862	0.029	0.807	0.921
Unpaid overtime	1.011	0.002	1.008	1.014	1.004	0.005	0.995	1.014
_cons					1.014	0.029	0.958	1.072

^a^Results retransformed to the original scale. For ease of interpretation, gender codes are reversed: male = 0; female = 1. CI, confidence interval; Coeff. coefficient; PE, experience proxy; SE, standard error; Separated/div/wid, separated/divorced/widowed.

Source: Work Outcomes Research Cost-benefit Survey 2005/06.

**Table 5:** **Blinder–Oaxaca decomposition of hourly wage rate differences between males and females stratified by occupation**^**a**^

	**Manager (*n *= 1,128)**				**Professional/technical (*n *= 6,151)**				**Clerical/service (*n *= 2,324)**			
	**Coeff.**	**Robust SE**	**95% CI**		**Coeff.**	**Robust SE**	**95% CI**		**Coeff.**	**Robust SE**	**95% CI**	
**overall**												
group_1 (males)	32.576	0.553	31.509	33.679	29.208	0.263	28.697	29.728	19.416	0.258	18.917	19.929
group_2 (females)	25.958	0.367	25.250	26.687	22.782	0.114	22.559	23.006	17.020	0.126	16.776	17.268
difference	1.255	0.028	1.202	1.310	1.282	0.013	1.256	1.308	1.141	0.017	1.107	1.175
explained	1.084	0.017	1.052	1.117	1.074	0.006	1.062	1.085	1.043	0.008	1.027	1.060
unexplained	1.158	0.021	1.117	1.200	1.194	0.012	1.172	1.217	1.093	0.015	1.065	1.123

^a^Results retransformed to the original scale. For ease of interpretation, gender codes are reversed: male = 0; female = 1. CI, confidence interval; Coeff., coefficient; SE, standard error.

Source: Work Outcomes Research Cost-benefit Survey 2005/06.

Referring to the individual endowments in the explained and unexplained sections of Table 4, coefficients greater than unity indicate a widening of the gap while coefficients less than unity indicate a closing of the gap. The explained component is the part of the outcome differential that is explained by group differences in the predictors. The unexplained part is usually attributed to discrimination but it also captures all potential effects of differences in unobserved variables [28]. Looking at the explained endowments, most of the differences between male and female employees relate to the local sector, clerical/service occupation, and number of children, followed by unpaid overtime. The trade/labour occupational variable helps to close the gap. Of particular interest is how much of the gender wage gap is due to differences in unpaid overtime. The results indicate that unpaid overtime explains 5% of the wage gap.

Regarding the unexplained component of the model, the gender differences in coefficients for females show that the gap is closed by most characteristics, as indicated by the value of the coefficients being less than unity. Work experience, however, leads to a substantial widening of the gap. The PE and PE^2^ variables show that as work experience rises, the wage gap increases at a decreasing rate. The intermittent nature of female employment was not captured in the model and this may have led to the overexaggeration of the work experience variable.

Further analysis was performed that included the interaction term between married and number of children (results not tabled). The raw gender wage differential, the explained and unexplained gaps remained unchanged (when results were rounded to three decimal points).

The decomposition model is also stratified by occupational group (Table 5). The unexplained components of the manager and professional categories are 15.8% and 19.4% respectively, compared with only a 9.3% gap within the clerical/service category.

## Discussion

This study identified that there are significant differences between males and females regarding their human capital endowments, job characteristics and family responsibilities that partly explained the gender wage gap. The results of the Blinder–Oaxaca decomposition revealed that, after adjusting for endowments, a gap of 16.7% remained unexplained.

This is in line with other Australian studies across all sectors that reported estimates of between 10 and 20% [9,11,39]. Stratifying the sample showed evidence of gender wage differentials that varied across occupation. Even so, males consistently earned disproportionately more than females across all occupational categories. The adjusted gender wage gap tended to be larger in higher-income occupational categories (for example, 19.4% in the professional/technical group) and smallest for the lower income occupation (for example, 9.3% in the clerical/services group). These findings are consistent with previous research [11].

Similar to previous studies [9,11,14], the returns on education were positive and increased with the level of education. Males received higher returns to schooling than females. Marital status was also an important determinant of earnings. Married males possessed a higher income than their single counterparts. These results are consistent with studies by Van Der Meer [14], Eastough and Miller [9] and Langford [32]. Married males’ greater attachment to paid employment may explain their relatively higher wages. In his paper on the sexual division of labour, Becker argued that the persistence of gender wage differences may arise from women’s greater responsibility towards informal unpaid work (for example, caring for children, the older person or those with physical disabilities, household chores, and so forth) [25]. Males under this arrangement are allocated the main financial responsibility of supporting the household. Married males are therefore likely to possess a greater commitment to paid employment compared with single males.

Similar to Miller [11] and Eastough and Miller [9], the analysis showed that married females earned less than unmarried females. The result that a greater number of children was associated with highe incomes may be due to the lack of information regarding the dependency of children; that is, older children may be more likely to have two working parents. Alternatively, a greater number of children in the household drive demand for higher income. Eastough and Miller found that only males with dependent children earned higher wages [9]. A limitation of the study is that no information on the age of the respondents’ children was collected. This would have facilitated stratifying the sample to assess any differences between new parents and parents of less dependent adolescents. Similar to others [11,40], we found that public-sector employees were better remunerated than their private-sector counterparts.

In the determination of hourly wages, years of experience became important for males but not for females. Females’ contribution towards household labour may perhaps limit their advancement within an organization and thus dampen the impact of experience on hourly wages. Another possibility is that the experience variable may be a better reflection of work experience for the male rather than the female sample. The data did not include information on actual labour market experience. Females’ intermittent workforce participation can result in potential experience being a poor measure of their actual experience. When the analysis was stratified by occupation, the work experience coefficients were not significant at all. Males possibly tend to progress as they gain experience and move out of lower paid occupations into manager roles. Across occupational groups, educational qualifications were important for higher ranks of occupational status, but the effect progressively disappeared in the lower ranks. This finding is consistent with previous research [6].

The descriptive statistics showed higher amounts of unpaid overtime performed by males. To ascertain the relationship between unpaid overtime and hourly wages, a log-lin regression was performed that controlled for confounding variables. When unpaid overtime was accounted for, the wage gender gap decreased slightly. Omitting unpaid overtime therefore tended to overestimate the wage gender gap. In addition, this study showed that females were less likely to work unpaid overtime than males, and that the wage effect for unpaid overtime for females was lower than that for males. That is, males were more likely to work unpaid overtime and receive higher incomes than females. This is in line with research from the Netherlands where it was found that the effect of unpaid overtime on wages was less for females than for males [14].

Miller [11] and others [9] have argued that the varying gender wage gap among the lower and higher income groups tends to reflect the methods by which pay is set. Consistent with their research, this study found a wider gender wage gap among higher-paid occupations. Among higher-paid occupations in the health sector (managerial and professional/technical) there is scope for bargaining and managerial discretion (for example, individual pay setting) to reward employees that signals their greater productivity. This is in contrast to the occupations with lower incomes (clerical/service), where enterprise bargaining and higher union coverage rates create fixed pay structures through collective agreements for the occupational group. This difference in pay determination may therefore go some way to explaining the wider gender wage gap within higher-earning occupations.

Workers with more complex job tasks and/or leadership roles may have a greater mismatch between paid work hours and actual work hours. Bell and Hart [36] showed that workers in occupations such as managers and professionals were more likely to report extra work for no pay. These workers tended to have a greater vested interest in the company, to have more responsibility and satisfaction in their work, may be less mobile and may be more difficult to replace. Nevertheless, male and females in these higher-paid occupations are likely to have the same level of vested interest, and therefore should have similar incomes. Workers in less skilled jobs, where work scheduling and job tasks are more precisely defined and demarcated, tend to be paid for all hours worked in excess of their standard work week. Meng [13] found that narrower gender wage gaps existed in firms that were easily able to identify labour productivity at the individual level.

A substantial gender wage gap existed within the professional/technical group. Female-dominated subcategories (such as the nursing profession) are likely to set their pay under a collective agreement. For instance, in Queensland 94% of nurses in 2005 were female [41] and most worked under collective agreements. Male-dominated subcategories, such as medical specialists, tend to work under an individual arrangement. Various studies confirm that pay determination significantly impacts on gender wage differentials [10-13]. The wage setting method of male-dominated and female-dominated subcategories and the subcategories themselves could not be identified. This is a limitation of the study. Another limitation is the use of broad occupational groupings. The estimate of the role of the occupational group would most probably increase if the model included more disaggregated information [42].

Studies that model gender differences in wages often extend the model to include job characteristics such as hours worked, occupation and industry [23]. Regarding the potential endogeneity of hours worked, our study deals with unpaid overtime in the Blinder–Oaxaca decomposition in a similar manner to previous studies of this nature that include hours worked [23,24]. Although a person may make more money because they work more unpaid overtime, or may work more unpaid overtime because they make more money, these effects are controlled for to some extent by the inclusion of job characteristics (occupation, supervision and sector) as control variables in the model. Furthermore, unpaid overtime was calculated by the actual minus expected hours worked per week. If an employee was expected by their employer to perform a certain amount of overtime, then actual and expected hours would equal each other. This further mitigated the endogenous nature of unpaid overtime. Although various studies use cross-sectional data to explain gender wage gaps, it is acknowledged that data from different periods would have been ideal since it would have produced unpaid overtime as a similar variable to the classic ones. A limitation of the study is the unavailability of data from different periods.

## Conclusions

The inclusion of the unpaid overtime variable is an important factor that appeared to be sensitive to the pay determination arrangements of the occupational category. Unpaid overtime should thus be included in any analysis of wages and incomes. However, the fact remains that, in the health sector, simply being female is the major contributing factor to the gender wage gap. Previous research shows that the primary cost of females earning less than males is the disincentive to work more hours [3,26]. The Australian health sector is expected to continue to face a shortage in skilled labour. Addressing wage differentials in this sector is one of several strategies that could be implemented to improve labour supply and meet the healthcare needs of an aging population.

## Endnotes

^a^For example, in contrast to the Work Outcomes Research Cost-benefit survey, the Australian Bureau of Statistics census reported certain groups of workers belonging to the Community and Personal Service sector rather than the Health and Community Services sector. The Community and Personal Service category includes workers that assist health professionals in the provision of patient care, provide information and support on a range of social welfare matters, and provide other services in the areas of aged care and childcare, education support, hospitality, defence, policing and emergency services, security, travel and tourism, fitness, sports and personal services [43].

^b^This concept of actual and expected is not unusual in the economics discipline. For example, economists often refer to actual and planned investment.

^c^In Queensland, children generally start school at 6 years of age.

^d^Various studies include additional variables in wage differential models such as duration of current employment [23,24], primary caregiver of dependent children, employment status of partner [23], union membership [24], and size of the organization [10,24]. Owing to data limitations, these variables were not included in our model.

## Abbreviations

HPQ: Health and Performance at Work Questionnaire

## Competing interests

The authors declare that they have no competing interests.

## Authors’ contributions

PAS conceived of the study. NV performed the statistical analysis and coordinated the manuscript. PAS and NV participated in its design. All authors helped to draft the manuscript. All authors read and approved the final manuscript.

## References

[B1] Kidd M Shannon M Convergence in the gender wage gap in Australia over the 1980s: identifiying the role of counteracting forces via the Juhn, Murphy and Pierce decomposition Appl Econ 2001 33 929 936

[B2] Sebastian S Gender Wage Gap Biggest in 28 Years 2011 UK: Commonwealth Bank of Australia

[B3] Cassells R Vidyattama Y Miranti R McNamara J The Impact of a Sustained Gender Wage Gap on the Australian Economy 2009 University of Canberra: National Centre for Social and Economic Modelling

[B4] Sicilian P Grossberg A Investment in human capital and gender wage differences: evidence from the NLSY Appl Econ 2001 33 463 471

[B5] Kidd M Goninon T Female concentration and the gender wage differential in the United Kingdom Appl Econ Lett 2000 7 337 340 10.1080/135048500351492

[B6] Nikolaou A Theodossiou I Returns to qualifications and occupation for males and females: evidence from the British workplace employee relations survey (WERS) 1998 Appl Econ Lett 2006 13 665 673 10.1080/13504850500401981

[B7] Daly A Kawaguchi A Meng X Mumford K The gender wage gap in four countries Econ Rec 2006 82 165 176 10.1111/j.1475-4932.2006.00313.x

[B8] Gregory R Labour market institutions and the gender pay ratio Aust Econ Rev 1999 32 272 278

[B9] Eastough K Miller P The gender wage gap in paid and self employment in Australia Aust Econ Pap 2004 43 257 276 10.1111/j.1467-8454.2004.00229.x

[B10] Kee H Glass ceiling or sticky floor? Exploring the Australian gender pay gap Econ Rec 2006 82 408 427 10.1111/j.1475-4932.2006.00356.x

[B11] Miller P The role of gender among low paid and high paid workers Aust Econ Rev 2005 38 405 417 10.1111/j.1467-8462.2005.00383.x

[B12] Voon D Miller P Undereducation and overeducation in the Australian labour market Econ Rec 2005 81 S22 S33 10.1111/j.1475-4932.2005.00247.x

[B13] Meng X Gender earnings gap: the role of firm specific effects Labour Econ 2004 11 555 573 10.1016/j.labeco.2003.09.006

[B14] Van der Meer P Is the gender wage gap declining in the Netherlands? Appl Econ 2008 40 149 160 10.1080/00036840600749557

[B15] Bell L Freeman R The incentive for working hard: explaining hours worked differences in the US and Germany Labour Econ 2001 8 181 202 10.1016/S0927-5371(01)00030-6

[B16] Landers R Rebitzer J Taylor L Rate race redux: adverse selection in the determination of work hours in law firms Am Econ Rev 1996 86 3229 3248

[B17] Verhoogen E Trade, quality upgrading, and wage inequality in the Mexican manufacturing sector Q J Econ 2008 123 489 530 10.1162/qjec.2008.123.2.489

[B18] Kremer M The O-Ring theory of economic development Q J Econ 1993 108 551 575 10.2307/2118400

[B19] Akerlof G Labor contracts as partial gift exchange Q J Econ Rec 1982 97 543 569 10.2307/1885099

[B20] Hashimoto M Firm-specific human capital as a shared investment Am Econ Rev 1981 71 475 482

[B21] Blinder A Wage discrimination: reduced form and structural estimates J Hum Resour 1973 8 436 455 10.2307/144855

[B22] Oaxaca R Male–female wage differentials in urban labor markets Int Econ Rev 1973 14 693 709 10.2307/2525981

[B23] Gibb S Fergusson D Horwood L Sources of the gender wage gap in a New Zealand birth cohort Austr J Labour Econ 2009 12 281 298

[B24] Watson I Decomposing the gender pay gap in the Australian managerial labour market Austr J Labour Econ 2010 13 49 79

[B25] Becker GS Human capital, effort, and the sexual division of labor J Labor Econ 1985 3 S33 S58 10.1086/298075

[B26] Cavalcanti T Tavares J The Output Cost of Gender Discrimination: A Model Based Macroeconomic Estimate, Volume 6477 2007 London: Centre for Economic Policy and Research

[B27] Commission P Australia’s Health Workforce 2005 Canberra: Australian Government

[B28] Jann B A Stata Implementation of the Blinder–Oaxaca Decomposition. ETH Zurich Sociology Working Papers No 5 2008 Zurich: Swiss Federal Institute of Technology Zurich

[B29] Gosse M The Gender Pay Gap in the New Zealand Public Service. Working Paper Number 15 2002 Wellington: State Services Commission

[B30] Withers G Hancock K, Sano Y, Chapman B, Fayle P Ohashi and Chapman and Millers papers: comment Japanese and Australian Labour Markets: A Comparative Study 1983 Canberra: Australia–Japan Research Centre 264

[B31] Chapman B Miller P Hancock K, Sano Y, Chapman B, Fayle P Determination of earnings in Australia: an analysis of the 1976 census Japanese and Australian Labour Markets: A Comparative Study 1983 Canberra: Australia–Japan Research Centre, Canberra 228 259

[B32] Langford M The gender wage gap in the 1990s Aust Econ Pap 1995 34 62 85 10.1111/j.1467-8454.1995.tb00017.x

[B33] Hilton MF Whiteford HA Sheridan JS Cleary CM Chant DC Kessler RC Wang PS The prevalence of psychological distress in employees and associated occupational risk factors J Occup Environ Med 2008 50 746 757 10.1097/JOM.0b013e31817e9171 18617830

[B34] Health and Performance at Work Questionnaire http://www.hcp.med.harvard.edu/hpq/

[B35] Australian Bureau of Statistics National Health Survey 2005 Basic, Confidentialised Unit Record File (CURF) No. 2079 2007 Canberra: Australian Bureau of Statistics

[B36] Bell D Hart R Unpaid work. Economica 1999 66 271 290

[B37] Kidd M Shannon M The gender wage gap: a comparison of Australia and Canada Ind Labor Relat Rev 1996 49 729 746 10.2307/2524519

[B38] Halvorsen R Palmquist R The interpretation of dummy variables in semilogarithmic equations Am Econ Rev 1980 70 474 475

[B39] Borland J The equal pay case – thirty years on Aust Econ Rev 1999 32 265 272 10.1111/1467-8462.00114

[B40] Papapetrou E The unequal distribution of the public-private sector wage gap in Greece: evidence from quantile regression Appl Econ Lett 2006 13 205 210 10.1080/13504850500393063

[B41] Australian Institute of Health and Welfare Nursing and midwifery labour force 2005 AIHW Health Labour Force Series No. 39. Volume Cat HWL40 2008 Canberra: Australian Institute of Health and Welfare 29

[B42] Chapman B Mulvey C An analysis of the origins of sex differences in Australian wages J Ind Relat 1986 28 504 520 10.1177/002218568602800402

[B43] Australian Bureau of Statistics ANZSCO – Australian and New Zealand Standard Classification of Occupations 2006 1 Canberra: Australian Bureau of Statistics

